# Phytochemical evaluation and exploration of some biological activities of aqueous and ethanolic extracts of two species of the genus *Plantago* L.

**DOI:** 10.1371/journal.pone.0298518

**Published:** 2024-02-29

**Authors:** Anouar Bouali, Ylenia Spissu, Antonio Barberis, Angela Fadda, Emanuela Azara, Germano Orrù, Alessandra Scano, Giuseppe Palmieri, Guy D’hallewin, Héla El Ferchichi Ouarda

**Affiliations:** 1 Laboratory of Plant Toxicology and Environmental Microbiology, Department of Life Sciences, Faculty of Sciences of Bizerte, University of Carthage, Carthage, Tunisia; 2 Institute of Sciences of Food Production, National Research Council, Sassari, Italy; 3 Institute of Biomolecular Chemistry, National Research Council, Sassari, Italy; 4 Department of Surgical Sciences, Molecular Biology Department, University of Cagliari, Cagliari, Italy; 5 Institute for Genetic and Biomedical Research, National Research Council, Sassari, Italy; University of Sassari: Universita degli Studi di Sassari, ITALY

## Abstract

*Plantago major* L. and *Plantago lagopus* L. are cosmopolitan species, belonging to the Plantaginaceae family, used in traditional and modern medicine. In this study, a phytochemical evaluation of different aqueous and ethanolic extracts of leaves and roots of both species from the region of Beja in Tunisia was performed. Some biological activities, including antioxidant, anticancer and antibacterial were also done. LC-MS qualitative analysis revealed that the aqueous extracts of the roots of *P*. *lagopus* were richer in polyphenols, mainly flavonoids (Luteoline 7-rutinoside, Luteoline 7-rhamnoside) and hydroxycinnamic acids including caffeic acid, than the hydro-ethanolic extracts. Additionally, we identified for the first time the presence of salicylic acid in the hot aqueous extracts of roots of *P*. *lagopus* and its absence in the roots of *P*. *major*. The antioxidant activity of the extracts was assessed using cyclic voltammetry (CV), revealing that the voltammograms of leaf and root extracts from *P*. *lagopus* exhibited a higher antioxidant capacity compared to those of *P*. *major*. Antiproliferative activity, was determined against two-colon cancer cell lines, demonstrated that only the 12 h treatments with *P*. *lagopus* leaf and root aqueous and hydro-ethanolic extracts at low concentration were able to significantly reduce the colon carcinoma coli-2 (CaCo-2) cells proliferation. The antibacterial /antibiofilm activity was performed on yeast, Gram- negative and +positive bacterial strains. We demonstrated for the first time that ethanolic extracts of leaves and roots of *P*. *lagopus* have an inhibitory activity against *Escherichia coli* and *Klebsiella pneumonia* at MIC = 2 μg/mL for leaves and 4 μg/mL for roots.

## 1. Introduction

The *Plantago* genus belongs to the Plantaginaceae family include 483 perennial and annual species distributed throughout the world comprising more than 200 plants with functional characteristics and/or disease healing effects [1, 2]. Plantaginaceae have a vast ecological range; being weeds of both arable and grasslands with a broad range of species and ecotypes worldwide-distributed [3–5]. Among the many species, mainly *Plantago major* L. and *Plantago lagopus* L. were investigated for their phytochemical content, and *P*. *major* has been highlighted for its richness in phenolic compounds, mainly flavonoids and phenolic acids [6–9]. While, iridoid and phenyethanoid glycosides are metabolites isolated from *P*. *lagopus* L. with nutritional and allelopathic properties [10–14]. In addition, it was reported that verbascoside and calceorioside A were present in leaf extracts of *P*. *lagopus* L [15]. Both species own several biological properties: among them, a notable antioxidant activity measured by the DPPH assay [16–20], and by the nitric oxide one [21, 22]. Antiproliferative studies on *P*. *major* L. leaf extracts evidenced an inhibition effect on the growth of MCF-7 breast cancer cells, UACC-62 melanoma cells [16], OVCAR, UISO cells [7], H 400 oral epithelial cells (OEC) [23] while, other studies have asserted the inhibitory activity against Ehrlich ascites tumors in mice [24]. Meanwhile, traditional medicine practices have been validated by scientific reports concerning the inhibitory activity of *P*. *lagopus* L. against Human Epidermal Carcinoma of the Larynx (HEP-2) and Human Rhabdomyosarcoma (RD) cell lines [15, 16]. Concerning antibacterial activity, *P*. *major* L. leaf extracts were reported to inhibit *Candida albicans*, *Saccharomyces cerevisiae* [25], *Porphyromonas gingivalis* [26], *C*. *glabrata*, *Staphylococcus aureus*, and *Pseudomonas aeruginosa* [27].

Concerning scientific reports, almost all researches on *P*. *major* have been addressed to shed light on plant areal organ properties (leaf and seed) while, practically no papers deal with roots. Concerning *P*. *lagopus*, in general, information is scarce on both bioactivity and antibacterial properties. Therefore, the objective of this study was to investigate the antioxidant, antiproliferative, and antibacterial properties of root and leaf extracts obtained from *P*. *major* and *P*. *lagopus*, collected in Tunisia, while also comparing the phytochemical profiles of these extracts.

## 2. Materials and methods

### 2.1. Chemical reagents

Gallic acid (GA; 3, 4, 5-trihydroxybenzoic) and ethanol (EtOH) were purchased from Sigma-Aldrich (Milan, Italy). Phosphate buffered saline (PBS) was prepared from NaCl (137 mM), NaOH (2.7 mM), Na2HPO4 (8.1 mM), and KH2PO4 (1.47 mM) and adjusted to pH 7.4. Dulbecco’s Modified Eagle Medium (DMEM), EMEM (Minimum Essential Medium Eagle with Earle’s Balanced Salt Solution), penicillin, streptomycin, sodium pyruvate, non-essential amino acids, glutamine, fetal bovine serum (FBS), Crystal violet, paraformaldehyde and acetic acid were purchased from Euroclone S.p.A. (Pero, Milan, Italy). Caco-2 and RKO cells were provided by the European Collection of Cell Cultures (ECACC, Salisbury UK). Bacterial strains were purchased from DSMZ (German Collection of Microorganism and Cell Cultures, Braunschweig, Germany).

### 2.2. Plant material

Leaves and roots of *P*. *lagopus* and *P*. *major* were collected in March 2022, before their flowering period when the highest concentration of bioactive compounds are metabolized, in the Tunisian region of Beja (Latitude: 36°43′32″ North; Longitude: 9°10′54″ East; Altitude: 248 msl) characterized by a calcareous-vertisol soil [28]. Whole plants kept cold from field to laboratory, at arrival in the laboratory were rinsed immediately, gently wiped with blotting paper and dissected separating roots and leaves and discarding stems. Then, roots were chopped into small pieces (1–2 cm) and together with the leaves dried at room temperature (23 ± 2 °C), in a dark and well-ventilated place, to prevent depletion of bioactive compounds by light or heat. Drying lasted till no weight changes occurred between two subsequent weightings. This condition was reached after 15 and 25 days for leaves and roots, respectively. Then the dried plant organs were finely grinded with a blade mixer, sieved, obtaining fine uniform sized matrixes (Ø = 250 μm) that were cold-stored (-80 °C) in sterile vacuum bags until further use.

### 2.3. Preparation of the extracts

Ten g of *P*. *lagopus* and *P*. *major* leaf and root powders were dispersed in 100 mL of distinct solvents, for different times: i) cold water for 24 hours; ii) hot water at 50 °C for 30 minutes; iii) hydroethanolic solution with 20, 40 or 80% of EtOH, for 2 h at 20 °C. After maceration, the solid matrix was withdrawn by a sequential filtering through gauze, glass fiber, and whatman filter paper using a Büchner funnel. Then, centrifugation at 3220 x *g* for 15 min took place to remove additional debris. For hydro-ethanolic extracts, the resulting supernatant was transferred to a rotavapor to remove ethanol by vacuum at 40–45 °C. Then, all aqueous extracts were frozen at -80 °C and freeze-dried. The dry residue was weighed, transferred in sealed vials, and stored at -80 °C until use.

The extraction yield (*R*) was calculated as percentage of the plant dry matter (DM) according to the formula:

$$\text{R}\;\left(\%\right)=\text{Mex}/\text{Mmv}*100$$

where Mex is the dry residue following the freeze-drying of the extract and Mmv is the mass of the starting dry plant matter.

### 2.4. LC-MS analysis

The LC–MS analysis was performed with an Agilent 1100 LC System (Agilent Technologies, Palo Alto, CA, USA) equipped with a binary pump, diode-array detector, column thermostat, degasser and an autosampler mode HTS-PAL. The LC was coupled to a single stage quadrupole mass spectrometer (Agilent G1946 MSD 1100) interfaced with an electrospray atmospheric pressure ionization source. LC–MS analysis was carried out to identify leaves and roots phenolic compounds from the two species in aqueous and ethanolic extracts. All analyses were performed in triplicate. Analytical data were acquired by Agilent ChemStation HP A.10.02. Chromatographic separation of phenolic compounds was carried out according to previous work with slight modifications [29]. A gradient program was employed using Eluent A (0.2% acetic acid– 0.1% trifluoracetic acid in water) and Eluent B (acetonitrile) with the following linear gradient settings: at 0 min 90% A, at 20 min 80% A, at 38 min 68% A. The flow rate was set at 0.250 mL/min, the run time was 55 min and the column temperature 35 °C. Injection volume was 10 μL. The diode array detector was set at 280 and 320 nm. Chromatographic separation of anthocyanins was carried out with a Luna C8 column (150 mm _*_ 2.1 mm, 3 μm, Phenomenex, Torrance, CA, USA) provided with a security guard cartridge (C8, 4 _*_ 2 mm). The mobile phases were ‘A’ (0.2% acetic acid– 0.2% trifluoracetic acid in water) and ‘B’ (acetonitrile). The applied elution conditions for the mobile phase ‘B’ were: a linear gradient increase from 10% to 20% followed by a 32% steady-elution starting from 0→20 min and from 20→38 min, respectively. The flow rate was set at 0.3 mL/min and the column temperature was 37 °C. Injection volume was 50 μL. The diode array detector was set at 270 and 520 nm. Mass spectra were acquired using electrospray ionization in the positive (PI) and negative (NI) ionization mode with the following conditions: m/z range 270–800, ion spray voltage 3200 mV and fragmentor 85 eV (PI), ion spray voltage 3400 mV and fragmentor 50 eV (NI). After optimization, heated nebulizer parameter was set as follow: temp. 35 °C, nebulizer pressure 42 psig, and flow rate of drying gas 9.8 L/min. According to a previous work [30], the identification of polyphenolic compounds was carried out by means of their UV spectra, molecular weight and MS fragments. Calibration curve was performed with five concentrations of different extracts in duplicate (5.5–550 mg/L, R2 = 0.999).

### 2.5. Electrochemical characterization and antioxidant activity determination

The electrochemical characterization of *Plantago* extracts and the antioxidant activity (AAox) determination were achieved by cyclic voltammetry as previously reported with some modifications [31, 32]. Measures were acquired by screen-printed sensors purchased by GSI Technologies (Burr Ridge, IL, USA), consisting of a 5 mm carbon working electrode (WE), an Ag/AgCl pseudo reference electrode (RE), and a carbon auxiliary electrode (AE). Currents were recorded by Quadstat, a commercial four-channel potentiostat (eDaQ Quadstat, e-Corder 410 and Echem software, eDAQ Europe Poland, Warsaw Poland). Cyclic voltammograms (CVs) were obtained from -0.2 V to +0.8 V (vs. Ag/AgCl pseudo-RE) at a scan rate of 0.1 V/s. A first 70 μL aliquot, containing only PBS (used as a supporting electrolyte), was deposited on the WE in order to obtain a baseline current; then, 70 μL aliquots of a 2 mg/mL *Plantago* extract solution were deposited on the sensor surface thus obtaining the corresponding CV pattern. All the experiments were performed in triplicate.

A quantitative comparison among the CV patterns of leaves and roots extracts was performed by integrating the voltammograms. The area under curve (AUC) was calculated at +0.5 V and expressed in microcoulombs (μC), as previously reported [33]. The redox potential of +0.5 V was used as a threshold to detect the antioxidant capacity of *Plantago* extracts, while additionally +0.8 V, refers to the activity of polyphenols with low reducing power which, in this work, were not accounted as antioxidants as reported previously [34–36].

### 2.6. Radical scavenging activity assessment

The hydroxyl radical scavenging activity was assessed with the spin trapping method coupled with Electron Paramagnetic Resonance (EPR) spectroscopy according to previous works [37, 38]. The hydroxyl radicals were produced with the Fenton reaction using a Fe (II) quinolic acid complex (0.1 mM and a ligand to metal ratio of 5:1) as Fe(II) source. Fe(II) reacted with hydrogen peroxide to produce hydroxyl radicals that were trapped with the nitrone spin trap DMPO. Diluted water solutions of the extracts were used to evaluate the hydroxyl radical scavenging activity. The results were expressed as EC_50_ (μg/mL). Experiments were performed at room temperature with a Bruker EMX spectrometer operating at the X-band (9.4 GHz) and a Bruker Aqua X capillary cell. EPR spectra were recorded immediately after the preparation of the reaction mixture. The concentration of the DMPO-OH adduct was estimated by double integration of the spectra. Three replicates were performed for each extract. The EPR instrument was set under the following conditions: modulation frequency 100 kHz, modulation amplitude 1 G, receiver gain 1 x 10^5^, microwave power 20 mW.

### 2.7. Cell culture

#### 2.7.1. Cell lines

CaCo-2 and RKO colon cancer cells were purchased from ATCC. CaCo-2 cells were cultured in Dulbecco’s modified Eagle’s medium (DMEM) high glucose supplemented with 10% Fetal Bovine Serum (FBS), 1% penicillin, 1% streptomycin, 1% non-essential amino acid and 1% sodium pyruvate, and then incubated at 37 °C under humidified atmosphere of 95% air and 5% CO_2_. RKO cell lines were grown in EMEM (Minimum Essential Medium Eagle with Earle’s Balanced Salt Solution) supplemented with 10% Fetal Bovine Serum (FBS), 1% penicillin, 1% streptomycin, 1% glutamine, 1% non-essential amino acid and 1% sodium pyruvate, and then incubated at 37 °C under a humidified atmosphere of 95% air and 5% CO_2_.

#### 2.7.2. Crystal violet test

RKO cells were plated at a density of 1 x 10^4^ in a 96 wells plate. CaCo-2 cells were plated at a density of 1 x 10^5^ in a 24 wells plates. After 24 h the cells were treated with increasing concentrations of *Plantago* extracts (0.1-1-10-25-50-100-150-250 μg/mL) and incubated for 12, 24 and 48 h.

After incubation, the medium was aspirated and the CaCo-2 and RKO cells viability was determined by the crystal violet staining assay, as described in previous studies with some modifications [39]. Cells were washed with PBS after being fixed with 4% paraformaldehyde. After 20 min the formaldehyde was removed, and the cells were stained with 0.1% crystal violet. Then, the crystal violet was removed, the cells were washed twice with PBS and solubilized with 10% acetic acid. Finally, the optical density of the studied solutions was measured at a wavelength of 595 nm using a Tecan Infinite M1000 PRO microplate spectrophotometer.

The percentage of cell viability was calculated as follows:

$$\%=\left(\text{optical}\;\text{density}\;\text{of}\;\text{treated}\;\text{cells}\right)/\left(\text{optical}\;\text{density}\;\text{of}\;\text{untreated}\;\text{cells}\right)*100$$

### 2.8. Evaluation of antibacterial activity

Antimicrobial activity evaluation of the *Plantago* species was performed according to the procedures described by the Clinical and Laboratory Standards Institute (CLSI) on *Bacillus subtilis*, *Staphylococcus aureus* and *Streptococcus mutans* (Gram +); *Escherichia coli*, *Pseudomonas aeruginosa* and *Klebsiella pneumonia* (Gram -) and one yeast, *Candida albicans*. A first line of evaluation was performed with the agar diffusion test [40]. This procedure was useful for the rapid assessment of bacterial resistance or susceptibility to the evaluated *Plantago* extracts. For each bacterial strain, 20 mL of agarized agar medium (Microbiol, Uta, Cagliari, Italy) at 55 °C was added to a 90-mm Petri dish, and, before the agar solidifies, four sterile iron rivets, 10 mm in diameter and 2 mm thick (Firm, Milan, Italy), was inserted into the agar mixture and then removed from the medium once cold. Under these conditions, each well can contain 50 μL of solution of the tested compound. Each strain was inoculated onto the surface of the plate using a sterile buffer with a standardized bacterial inoculum of 5 x 10^7^ colony forming units (CFU). Three wells were used for each compound test and two for the negative control. Petri dishes were incubated in air at 37 °C for 24 h for aerobic strains and in 5% CO_2_ at 37 °C for microaerophilic species. After incubation, the diameter of the inhibition halo was measured. The experiment was performed in triplicate. The diameter of inhibition is proportional to the logarithm of the concentration [mol/L] of the compound. Broth dilution and antibiofilm tests were performed only for compounds that showed activity.

#### 2.8.1. Minimum inhibitory concentration and minimum bactericidal concentration test

Minimum inhibitory concentration (MIC) and minimum bactericidal concentration (MBC), respectively, were determined by the microdilution method. This evaluation was performed in sterile 96-well microplates, and each well contained serial dilutions (50 to 0.04%) of each compound dissolved in nutrient broth. Briefly, concentrations between 4 mg/mL and 0.039 mg/mL of each extract were tested, and the final concentration of the strains was 1 x 10^7^ CFU/mL. The experiment was repeated three times. After 24–48 h of incubation at 37 °C in an appropriate atmosphere (air or 5% CO_2_), the MIC was the lowest concentration of the tested compound that inhibit visible growth, i.e., that show the same absorbance (620 nm) as the negative control, measured with a Multiskan FC microplate photometer (ThermoFisher Scientific IT, Milan, Italy). Differently, MBC represents the lowest concentration capable of killing 99.99% of the initial inoculum (CFU/mL) when microbial suspensions was placed in agar medium.

#### 2.8.2. Anti-biofilm assay

Determination of the minimum inhibitory concentration of biofilm (MICB) was performed according to the crystal violet staining protocol described by the Montana University Center for Biofilm Engineering (http://www.biofilm.montana.edu) with some modifications. Therefore, after 48 h of incubation, soil was aspirated from the plate in which the MIC was evaluated. The biofilm adhering to the surface of the wells was stained with 100 μL of crystal violet (4%) and left for 20 min. The crystal violet was removed and washed with 100 μL NaCl and solubilized with 200 μL acetic acid (30%). Finally, the absorbance of the biofilm was measured at 620 nm with a Multiskan FC microplate photometer (Thermo Fisher Scientific IT, Milan, Italy).

### 2.9. Statistical analysis

The statistical analysis was performed by GraphPad Prism 5 for Windows software (Graph-Pad Software, Inc., La Jolla, CA, USA). Phenolics content of the extracts from the two *Plantago* spp. was expressed as mg/g of dry matter (DM). AAox was expressed as micromoles equivalents of gallic acid/g DM. For analytical tests, a one-way ANOVA was performed to compare results obtained with different analytical methods, using a unifactorial complete randomized block design. Mean comparisons were calculated by Fisher’s least significant difference (LSD) test at p ≤ 0.05. Where not otherwise specified, biological tests were repeated three times. A one-way ANOVA was performed to highlight significant differences among treatments. The Student–Newman–Keuls (SNK) test was used to separate the mean values (p ≤ 0.01). The mean value ± standard deviation (SD) was reported in the figures.

## 3. Results

### 3.1. Extraction yield

Extraction is a fundamental step in the isolation and recovery of phytochemical compounds of plant origin [41, 42]. According to the results presented in Table 1, species and solvent used influenced the extraction yield. We observed significant differences in extraction yield when using water and ethanol for both species and both plant parts for *P*. *major* (p ≤ 0.05). However, for *P*. *lagopus*, there were no significant differences between leaf and root extracts. Despite of a long contact time between the matrix and the solvent, the extraction in water at 20 °C provided the lowest yields of dry residue. On the other hand, the highest recovery of dry residue occurred by the hot water (50 °C) extraction for 30 min. The two-hour extractions with hydro-ethanolic solvents were effective but still the recovered dry residue resulted lower than by hot water (Table 1). Among the hydro-ethanolic solutions, the extraction with the 40% ethanol was the most effective. In this work, by heating the water the highest quantity of water-soluble metabolites was recovered in the shortest extraction period (30 min). The lowest yields with the longest extraction durations occurred with cold-water (24 h), faster but with intermediate yields occurred by employing the ethanol solutions (120 min).

**Table 1:** Extraction yield (% of DM) from different organs of *P*. *lagopus* and *P*. *major* ^x^.

Species	Organ	Water (20°)	Water (50°)	Ethanol (%)		
				20	40	80
*P*. *lagopus*	Leaves	6.34 d^Y^	22.49 a	14.23 b	14.41 b	11.10 c
	Roots	5.64 e	16.35 a	10.40 d	14.02 b	11.45 c
*P*. *major*	Leaves	2.91 e	12.03 a	4.22 d	6.82 b	4.99 c
	Roots	3.89 d	10.93 b	7.08 c	12.69 a	9.90 b

^x^ dry residue expressed as % of dray matter (DM) subjected to extraction

^Y^Means in rows followed by unlike letters differ significantly by Fisher’s LSD procedure, p ≤ 0.05.

In accordance with the extraction efficiency results, reported in Table 1, it was decided to exclude from further experimentation the extraction with cold water and the one at 20 and 80% EtOH. Thus, the chemical and electrochemical characterization, as well as the biological assays on the cell cultures, were performed only for the extracts attained with hot water and 40% EtOH.

### 3.2. LC-MS qualitative result of the different extracts

Phenolic compound identification of the extracts of the investigated species was performed by LC-MS. The results evidenced that leaves and roots of both species are rich in polyphenols and the main compounds of each extract are reported in Table 2. The complete list of detected polyphenols, obtained with different extraction methods, from leaves and roots of *P*. *lagopus* and *P*. *major* is provided as S1 and S2 Tables in S1 File, together with the LC-MS chromatogram of each extract (S1-S4 Figs in S1 File). Dicaffeoylquinic acid was detected in all extracts of *P*. *major*, while Quercetin 7- rutinoside was found only in the ethanolic extracts of leaves and roots. Melittoside was detected in ethanolic extracts from *P*. *major* leaves. Concerning acteoside (verbascoside) and plantamajoside both were detected in all extracts from *P*. *lagopus* leaves and roots. Hot water extract of *P*. *lagopus* roots resulted richer than the hydro-ethanolic ones in polyphenols, mainly flavonoids (luteoline 7-rutinoside, luteoline 7- rhamnoside) and hydroxycinnamic acids including caffeic acid. In addition, 2’-Acetyl- campneoside, coumaroylic acid di-hexoside and luteoline-4’-o-glucoside were detected in leaf extracts. Salicylic acid (SA, 2-hydroxybenzoic acid) was identified for the first time in the roots of *P*. *lagopus*.

**Table 2:** Phytochemicals detected by LC-MS in heated-water and hydro-ethanolic leaf and root extracts of *P*. *major* and *P*. *lagopus*. ^X^ ^Y^.

Species	Organ	Extract	RT (min)	Molecular formula	m/z experimental	Compound
*P*. *major*	Leaf	Water (50 °C)	43,31	C_25_H_24_O_12_	515,12021	Dicaffeoylquinic acid
			47,69		1002,32083	ND^Y^
		EtOH (40%)	35,59	C_29_H_36_O_16_	639,18994	Plantamajoside
			41,91	C_27_H_30_O_16_	611,16098	Quercetin 7-rutinoside
			43,31	C_25_H_24_O_12_	515,12021	Dicaffeoylquinic acid
			47,69		1002,32083	ND
			49,13		1002,32106	ND
	Roots	Water (50 °C)	39,07	C_33_H_40_O_22_	789,21003	Quercetin3,7,4’-triglucoside
			43,31	C_25_H_24_O_12_	515,12021	Dicaffeoylquinic acid
			47,69		1002,32083	ND
		EtOH (40%)	41,91	C_27_H_30_O_16_	611,16098	Quercetin 7-rutinoside
			43,31	C_25_H_24_O_12_	515,12021	Dicaffeoylquinic acid
			46,39	C_26_H_28_O_16_	595,13697	Quercetin 3-arabinoside 7-glucoside
			47,08	C_31_H_40_O_17_	683,22103	2-Ethoxy plantamajoside
			47,69		1002,32083	ND
*P*. *lagopus*	Leaves	Water (50 °C)	23,11	C_16_H_18_O_9_	355,10092	Chlorogenic acid isomer
			36,00	C_29_H_36_O_16_	639,18744	Plantamajoside
			38,51	C_29_H_36_O_15_	625,21054	Acteoside
			40,89	C_21_H_20_O_11_	449,10580	Luteolin-4’-o-glucoside
			50,45		535,10575	Coumaroyl acid di Hexoside
		EtOH (40%)	36,00	C_29_H_36_O_16_	639,18744	Plantamajoside
			38,51	C_29_H_36_O_15_	625,21054	Acteoside
			40,89	C_21_H_20_O_11_	449,10580	Luteolin-4’-o-glucoside
			44,56		1130,38828	ND
			50,45		535,10575	Coumaroyl acid di Hexoside
	Roots	Water (50 °C)	29,53	C_29_H_36_O_17_	655,18257	Helicoside
			31,95	C_27_H_30_O_15_	595,16448	Luteolin 7-rutinoside
			32,30	C_21_H_20_O_10_	431,09854	Luteolin 7-rhamnoside
			36,00	C_29_H_36_O_16_	639,18744	Plantamajoside
			38,51	C_29_H_36_O_15_	625,21054	Acteoside
		EtOH (40%)	36,00	C_29_H_36_O_16_	639,18744	Plantamajoside
			38,51	C_29_H_36_O_15_	625,21054	Acteoside
			39,35	C_33_H_40_O_21_	773,2796	Quercetin 3-O-galactosyl-rutinoside
			42,89	C_23_H_26_O_11_	478,48924	Plantainoside A
			50,83	C_15_H_10_O_6_	287,05419	Luteolin

^x^ Only the 5 most represented compounds (those corresponding to the highest peaks in the chromatograms S1-S4 in S1 File) are reported for each extract

^Y^ ND: not determined

### 3.3. Electrochemical evaluation of antioxidant activity

**Fig 1:**
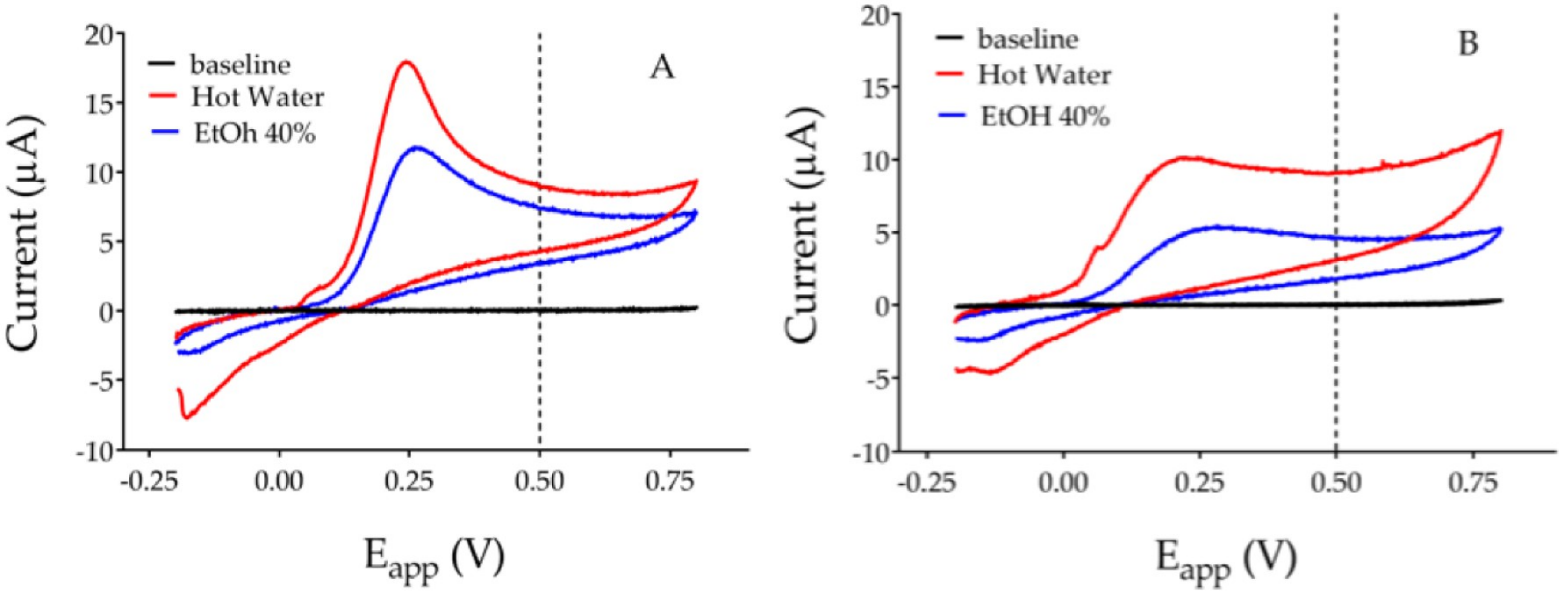
Cyclic voltammograms, with a scanned potential range (E_app_) comprised between -0.2 V and +0.8 V *vs* Ag/AgCl reference electrode, in the absence (black line) and in the presence of 2 mg/mL of aqueous (red line: Water 50 °C) and hydro-ethanolic (blue line: Ethanol 40%) *P*. *lagopus* leaf (A) and root (B) extracts.

**Fig 2:**
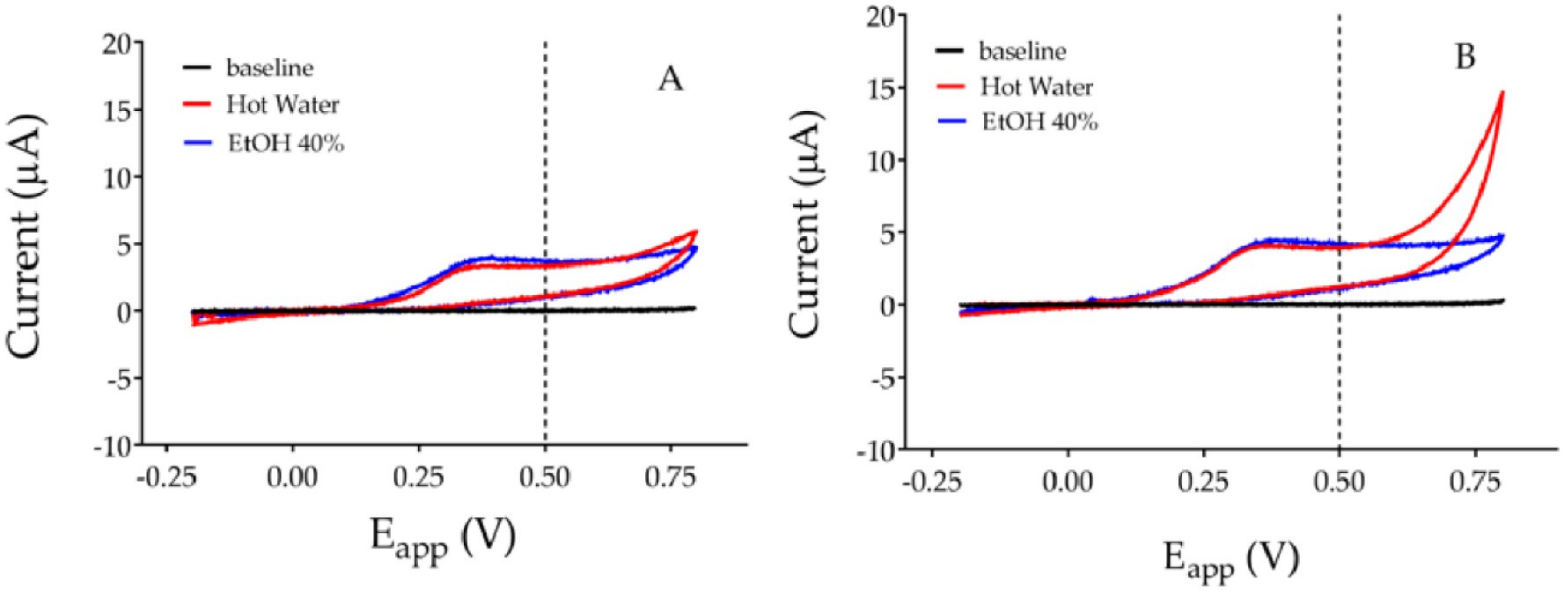
Cyclic voltammograms, with a scanned potential range (E_app_) comprised between -0.2 V and +0.8 V *vs* Ag/AgCl reference electrode, in the absence (black line) and in the presence of 2 mg/mL of aqueous (red line: Water 50 °C) and hydro-ethanolic (blue line: Ethanol 40%) *P*. *major* leaf (A) and root (B) extracts.

The electrochemical evaluation of antioxidant activity is based on the principle that the lower the ionization potential, the greater the antioxidant capacity of a molecule, or of a phytocomplex [33, 34, 36, 43, 44]. The electrochemical behavior of the extracts at different applied potential, was carried out by cyclic voltammetry, in accordance with previous studies [45]. The obtained voltammograms from the different extracts are in the Figs 1 and 2.

The voltammograms of *P*. *lagopus* leaf and roots extracts were different, regardless of the extraction solvent used (Fig 1). Aqueous and ethanolic extracts of leaves of *P*. *lagopus* showed voltammograms with similar shape (Fig 1A). Irrespective the quantity of phenolic compounds extracted, the voltammograms showed a peak between 0.23 and 0.27 V, thus indicating that similar classes of compounds were extracted.

The voltammograms of leaf and root extracts of *P*. *major* were also different: a well-represented phenolic component between 0.2 and 0.35 V contributes to the antioxidant activity of the extracts, even though significantly lower oxidation currents than *P*. *lagopus* were recorded (Fig 2). The shape of the voltammograms of *P*. *major* seems to be influenced more by the extraction solvent than by the yield: currents corresponding to a well-defined peak increased from 0.6 to 0.8 V in Fig 2B, only in the aqueous extracts.

Unlike shapes correspond to different AUCs reported, both at +0.5 V and +0.8 V, in Table 3. The AUC values at +0.5V refer to antioxidant activity while, AUC values at +0.8V estimate the total polyphenols content.

**Table 3:** Area under curve of CVs at +0.5 and +0.8 V applied potential of *P*. *lagopus* and *P*. *major* leaf and root extracts^X^.

Extraction method	E_app_ (V)	*P*. *lagopus*		*P*. *major*	
		Leaves	Roots	Leaves	roots
Hot water	+ 0.5	4.532 a (a)	3.977 b (a)	0.846 d (n.s.)	1.074 c (n.s.)
EtOH 40%	+ 0.5	3.245 a (b)	1.882 b (b)	0.995 c (n.s.)	1.162 c (n.s.)
Hot water	+ 0.8	7.129 a (a)	7.006 a (a)	5.866 b (a)	3.099 c (a)
EtOH 40%	+ 0.8	5.327 a (b)	3.291 b (b)	2.186 d (b)	2.429 c (b)

^X^ Means in rows followed by unlike letters differ significantly by Fisher’s LSD procedure, p ≤ 0.05. Means in column followed by (unlike) letters differ significantly by Fisher’s LSD procedure, p ≤ 0.05. n.s. = not significant

Figs 1 and 2 and Table 2 evidence that the leaf and root extracts of *P*. *lagopus* have higher antioxidant capacity than those of *P*. *major*. It is also clear that *P*. *lagopus* leaf and root aqueous extracts have higher antioxidant capacity than the hydro-ethanolic ones.

Lastly, all the obtained voltammograms did not show reduction peaks, thus indicating the irreversibility of the oxidation of the extracted polyphenols.

### 3.4. Antioxidant activity by spin trapping of hydroxyl radical coupled with electron paramagnetic resonance (EPR) spectroscopy

**Fig 3:**
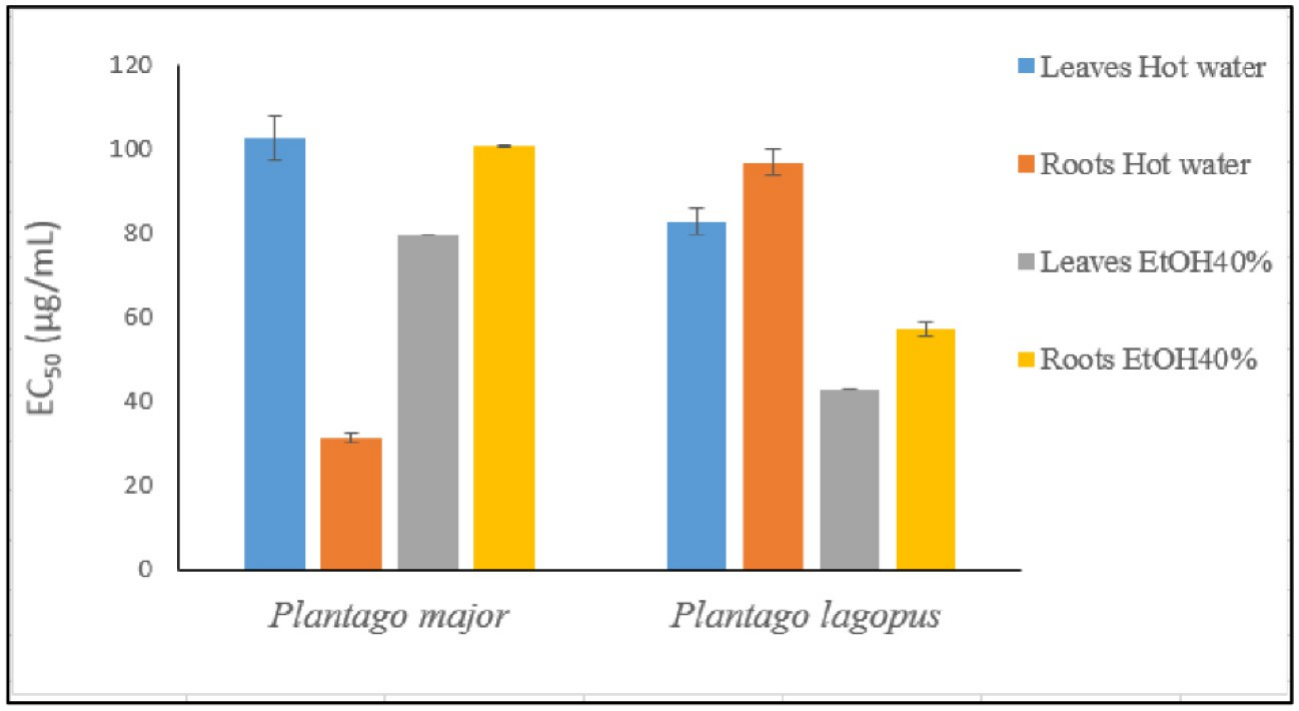
Hydroxyl radical scavenging activity of aqueous and hydro-ethanolic extracts of roots and leaves of *P*. *lagopus* and *P*. *major*. The values of EC_50_ are presented as mean ± standard deviation (n = 3 independent experiments).

The hydroxyl radical scavenging activity (HRSA) of water and ethanolic extracts of roots and leaves of *P*. *lagopus* and *P*. *major* is represented in Fig 3. The hot water extracts of *P*. *major* roots showed a higher HRSA than ethanolic ones (40% EtOH), being their EC_50_ 31.49 ± 1.29 and 100.95 ± 0.21 μg/mL, respectively. On the contrary, in the leaves, the ethanolic extracts displayed lower EC_50_ values (EC_50_ = 79.61 ± 0.01 μg/mL) than hot water ones (EC_50_ = 102.49 ± 5.23 μg/mL).

The extracts of *P*. *lagopus* showed a slightly lower HRSA than *P*. *major*. In particular, leaf hydro-ethanolic extracts showed the lowest EC_50_ value (42.92 ± 0.09 μg/mL) therefore the highest antioxidant power.

### 3.5. Antiproliferative activity

**Fig 4:**
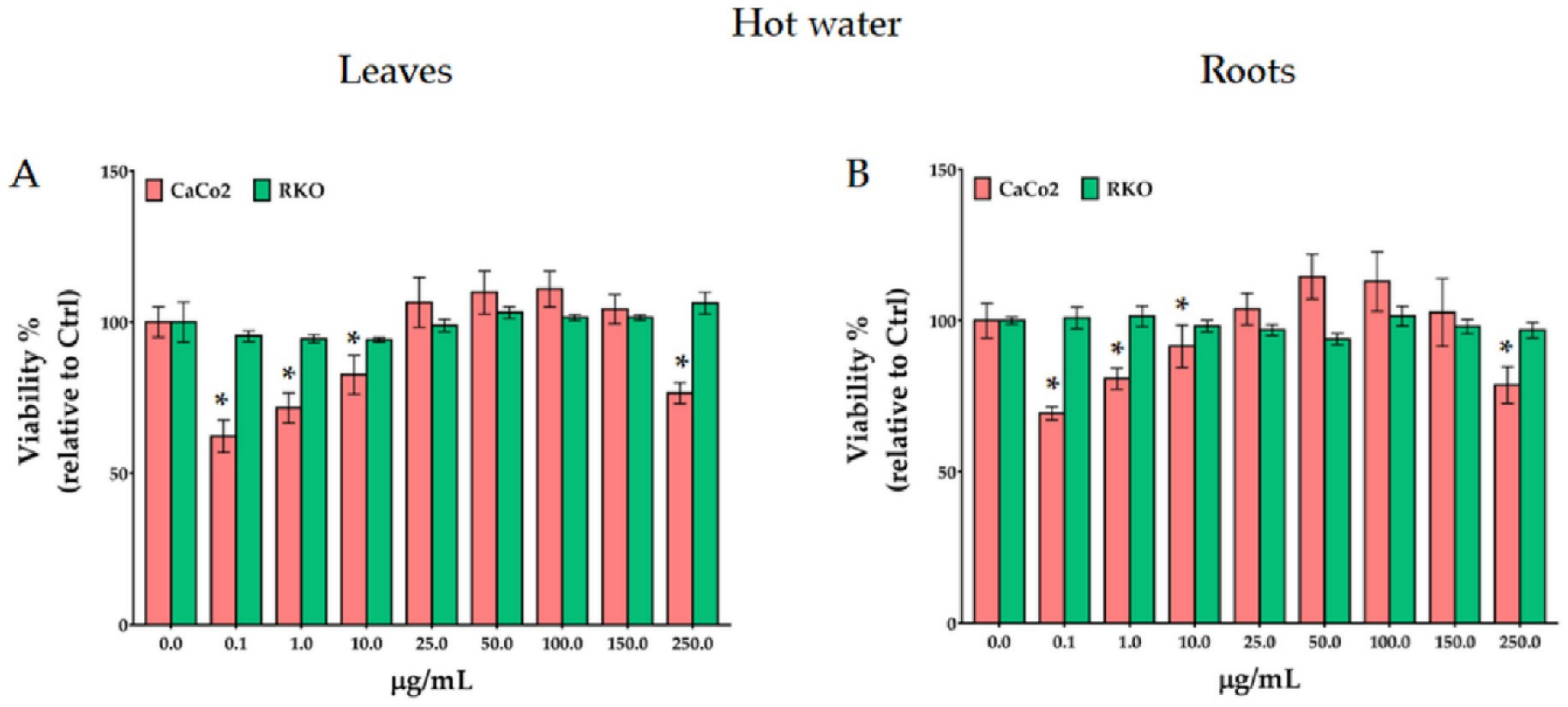
Effect of 12 h treatments with *P*. *lagopus* leaf (A) and root (B) extracts attained with hot water at 50 °C on viability of CaCo-2 and RKO cancer cells. *significantly different at *p* < 0.05.

**Fig 5:**
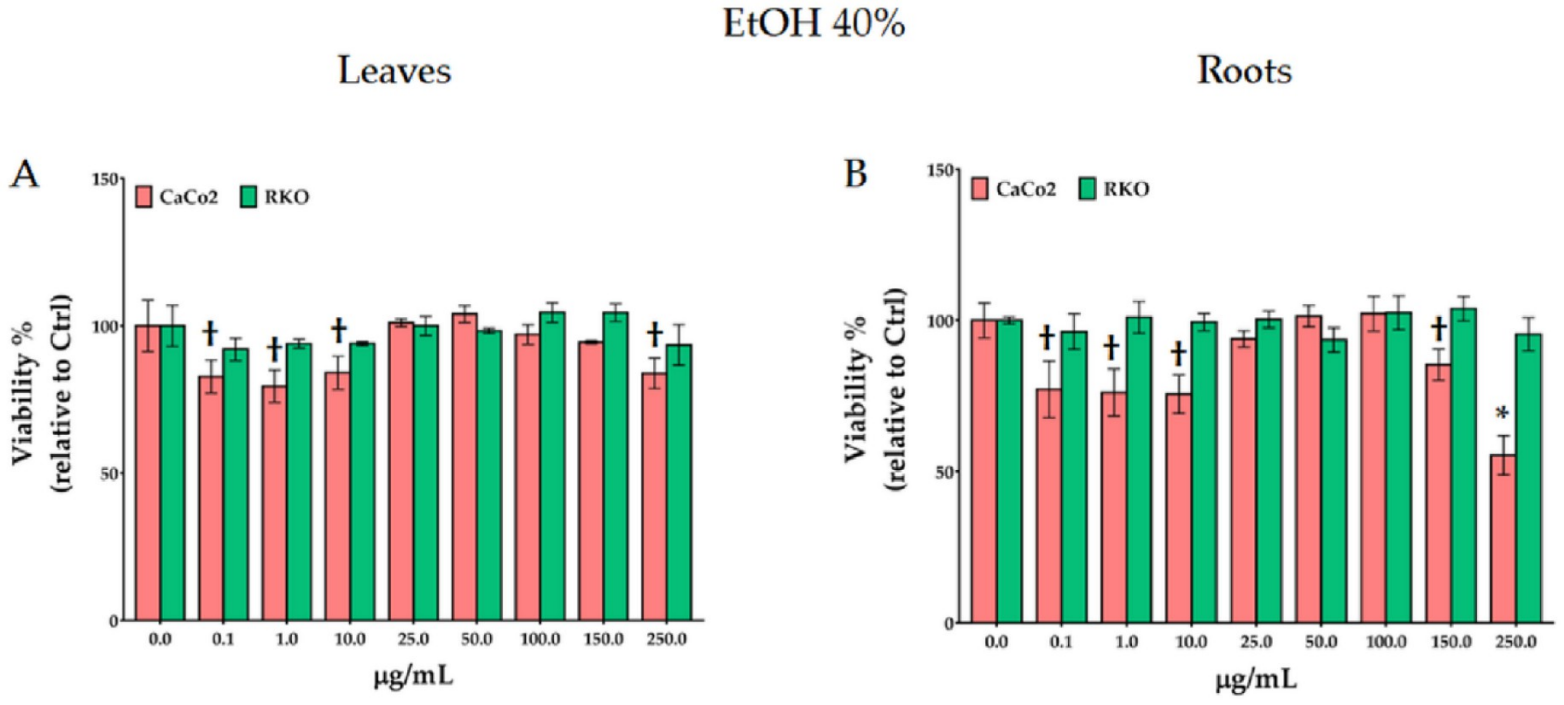
Effect of 12 h treatments with *P*. *lagopus* leaf (A) and root (B) hydro-ethanolic extracts on viability of CaCo-2 and RKO cancer cells. * †significantly different at *p* < 0.05 and *p* < 0.1, respectively.

To assess the effect of the treatments with *P*. *lagopus* and *P*. *major* extracts on colon cancer disease we selected two different cell lines, CaCo-2 (non-metastatic cells) and RKO (metastatic ones), using the crystal violet viability assay. The results reported in Figs 4 and 5. It evidence that only the 12h treatments with *P*. *lagopus* leaf and root aqueous and hydro-ethanolic extracts were able to significantly reduce the CaCo-2 cells proliferation. No significant effects were observed at 24–48 h (S5-S8 Figs in S1 File) and no notable effects were observed on RKO cells. *P*. *lagopus* hot water extracts of leaves (Fig 4A) resulted more effective compared to the root ones (Fig 4B), only on CaCo-2 cells, and low concentrations were more effective than high ones. Indeed, compared to the control, at 0.1 μg/ml the viability was lower (38% and 31% for leaf and root, respectively) with respect to 250 μg/mL (24% and 21% for leaf and root, respectively). The higher inhibition efficacy observed at low concentrations suggests a hormesis-like behavior, aligning with previous research [44, 46].

The same effect, even though less marked, was observed with the hydro-ethanolic extracts (Fig 5A and 5B). Cell viability decreased by low concentration (between 0.1 and 10 μg/mL). Whereas viability was not affected between 25 and 150 μg/mL and between 25 and 100 μg/mL for leaf and root extracts respectively. While, compared to control, root extract at 250 μg/mL reduced viability by a 45% (Fig 5B). The different efficiency between aqueous and hydro-ethanolic extracts highlighted by the crystal violet assay is consistent with the data obtained by cyclic voltammetry (Fig 1) which recorded a greater electrochemical activity in the extracts with hot water.

The treatments with *P*. *major* leaf or root extracts were not effective at any time (S9-S14 Figs in S1 File).

### 3.6. Antibacterial activity

The results of antibacterial activity clearly evidenced that hot water extracts of the different organs from the two species had no antimicrobial properties, nor on Gram+ or Gram-. In the present study, a strong inhibition activity was monitored by the ethanolic extracts of the two species with Gram- bacteria (Table 4).

**Table 4:** MIC, MBC and MBIC values attained for the tested Gram-negative bacteria following the amendment with 40% ethanolic extracts of two *Plantago* species ^X^ observed in the Gram-negative bacteria tested.

Species	*P*. *lagopus*						*P*. *major*					
Organs	Leaves			Roots			Leaves			Roots		
	MIC	MBC	MBIC	MIC	MBC	MBIC	MIC	MBC	MBIC	MIC	MBC	MBIC
*E*. *coli*	2 ^Y^	>4	2	4	>4	2	2	>4	2	2	>4	2
*Klebsiella pneumonia*	2	>4	2	4	>4	2	4	>4	>4	2	>4	2

^X^ Minimum Inhibitory Concentration (MIC); Minimum Bacterial Concentration (MBC); Minimum Bacterial Inhibition Concentration (MBIC).

^Y^ Concentration: μg/mL (dry residue)

It is interesting to note that between the ethanolic extracts a clear inhibition of *Escherichia coli* and *Klebsiella pneumonia* growth occurred when the media was amended with root or leave extracts of both species. Concerning the leave extracts of *P*. *lagopus* the same (2 μg/mL) concentrations for MIC and MBC were found while, root extracts were less effective (4 μg/mL). *P*. *major* extracts from leaves and roots evidenced a different behavior on the two bacteria with leave extract being lesser effective on *K*. *pneumonia* (MIC = 4 μg/mL) while, no difference was found for root extracts (Table 4). In addition, MIC values of *P*. *major* root extracts resulted lower than *P*. *lagopus* once (Table 4).

In addition, the tested ethanolic extracts of the two species showed an anti-biofilm effect towards *E*. *coli* and *K*. *pneumonia* and even at a low minimum biofilm inhibitory concentration (MIBC = 2 μg/mL). This encourages further research on *P*. *lagopus* extracts for antimicrobial treatments.

## 4. Discussion

Previous experiences demonstrated that the yield depends on several parameters such as solvent, pH, temperature, extraction time and sample composition [47]. The mixing of water and organic solvents results more sustainable compared to the use of sole organic solvents and can facilitate the extraction of plant metabolites, not only polyphenols but also protein or carbohydrates [48]. It was evident, by modifying the extraction variables, that high yields of polyphenols correlated positively with the extraction duration as well as the nature of the solvent (methanol > ethanol > water) [49]. Additionally, the same authors pointed out that negative effect could occur when extractions were long lasting with high-levels of organic solvents. Thus, this finding may support our feeble extraction efficiency attained with 80% EtOH compared that attained with 40% EtOH. Clearly, from an industrial point of view, the extraction protocol must take into account not only the efficiency but also the economics of the process thus, a compromise between yield, cost of the solvent, and/or the heating of the water at 50 °C.

Correspondingly, consistent with other results, leaf extracts of *P*. *major* contained gallic acid, vanillic acid, caffeine and quercetin [50]. In addition, in agreement with some findings [6, 51], chlorogenic acid isomers and caffeic acid derivatives, such as plantamajoside and acetoside were detected in the present ethanolic extracts. Dicaffeoylquinic acid was detected in all extracts of *P*. *major*, while Quercetin 7- rutinoside was found only in the ethanolic extracts of leaves and roots. Same chemical profile was also reported, where plantamajoside was identified in the ethanolic (50%) and aqueous extracts of *P*. *major* aerial parts [12, 23]. Plantamajoside, extracted from *P*. *major* leaves, has been shown to own antioxidant, anti-inflammatory, antibacterial and cytotoxic properties [6, 23, 52]. Concerning iridoid glycosides, Samuelsen, and Taskova et al. reported the presence of aucubin in extracts of *P*. *major* [6, 53]. This compound was not detected under the present LC-MS analytical conditions where different carrier solvents were employed, thus it cannot be excluded that traces were present in the examined extracts. While melittoside, which is an iridoid glycoside was detected in Tunisian *P*. *major* leaves and has been also isolated from the aerial parts of this species [54]. Acteoside (verbascoside) and plantamajoside were both detected in all extracts from *P*. *lagopus* leaves and roots. The hot water extract of *P*. *lagopus* roots were richer in polyphenols, such as luteolin 7-rutinoside and luteolin 7- rhamnoside, compared to the hydro-ethanolic extracts. These polyphenols were identified for the first time in the roots of this species. In addition, 2’-Acetyl- campneoside, coumaroylic acid di-hexoside, Helicoside, Martinoside and luteolin-4’-o-glucoside were detected in leaf extracts. Our results corroborate those of Beara et al. who identified luteoline-7-O glucoside and quercetin and the absence of apigenin and rutin [17]. It is noteworthy to report that salicylic acid (SA, 2-hydroxybenzoic acid) was identified in the roots of *P*. *lagopus* and not in those of *P*. *major*. According to the reports on the role of SA as a mobile signal of systemic acquired resistance (SAR) to biotic and abiotic stress in plants [55, 56], this result strengthen the finding of some authors who ranked *P*. *lagopus* among the most drought resistant *Plantago* species and *P*. *major* among the less ones [57]. The attained results for the extracts of *P*. *lagopus* agreed with those of Harput et al. and Gonçalves et al., who reported the presence of verbascoside in leaf extracts [15, 58]. Furthermore, the present findings are supported by the report of Gálvez et al. who identified phenylpropanoid glycoside, verbascoside, flavonoid, luteolin-7-O-β glucoside, aucubin and iridoid in the methanolic extract of *P*. *lagopus* aerial parts [59]. Concerning verbascoside, previous studies stated that antioxidant and cytotoxic characteristics of extracts were strictly dependent on the levels of this metabolite [15]. Later, other research disputed this statement on the positive correlation between the antioxidant activity and the content of verbascoside in the extracts [58]. Previous studies showed that methanolic extracts of *P*. *lagopus* containing luteolin-7-O-glucoside and its aglycone, luteolin, resulted cytotoxic on three cell lines [16, 60]. From the same species iridoid glycosides namely plantamajoside, luteolin-7-O-ß-D-glucopyranoside, chlorogenic acid and rosmarinic acid were isolated from methanolic extracts of the aerial parts [59].

In a recent study, the richness of *P*. *lagopus* ethanolic extracts in rutin, naringenin, quercetin, p-hydroxybenzoic acid, ellagic acid, vanillic acid, catechol, cinnamic acid, ferulic acid, benzoic acid and chlorogenic acid was provided [14]. Grubešić et al. in a comparative study among different species in Croatia, evidenced that methanolic extracts of *P*. *lagopus* leaves had the highest yield of phenolic acids (chlorogenic acid and caffeic acid) (0.116%) compared to the other studied species which contained 0.008 and 0.020% of quercitin and hyperoside, respectively [61].

According to Kilmartin et al. the anodic peak current is expected to be proportional to the concentration of the antioxidants [35]: unlike this, in this work no significant correlation was found between yield and AUC values. Differently, there is not significant dissimilarity between the antioxidant capacity of aqueous and hydro-ethanolic extracts of *P*. *major*.

In agreement with previous research, it was also observed that a large part of polyphenols ionized at a potential greater than 0.5 V and, therefore, they should not be accounted as antioxidants [36]. Accordingly with previous works [62, 63], these polyphenols should be included in the calculation of total polyphenols with a not-better specified activity. It was shown that caffeic acid isolated from *P*. *lanceolata* has a well-formed anodic peak, but the inductive effects of hydroxyl groups on the double bond, which was conjugated to the aromatic ring in caffeic acid, make the molecule more difficult to be reduced, therefore, the absence of a cathodic (reduction) peak of caffeic acid [64].

The results of the hydroxyl radical scavenging activity of water and ethanolic extracts of roots and leaves of *P*. *lagopus* and *P*. *major* agree with Saffidine et al. who observed, on *P*. *major* leaves, differences among the radical scavenging activities measured with the DPPH method, depending on the extraction solvent [65]. Moreover, it was reported that verbascoside and calceorioside A, isolated from the aqueous extracts of the aerial parts of *P*. *lagopus*, have significant free radical scavenging activity with IC_50_ = 21.71 and 22.45 μg/mL respectively [15]. All the obtained extracts contain acteoside (verbascoside), characterized by its antioxidant activities [15, 66, 67]. Therefore, it could be the responsible compound for this antioxidant activity, along with other phenolic compounds.

The obtained results do not find large confirmation in the literature due to the lack of investigation on aqueous or ethanolic extracts on the genus *Plantago*. Differently, the antioxidant properties of methanol extracts of selected *Plantago* species (*P*. *argentea* Chaix., *P*. *holosteum* Scop., *P*. *major* L., *P*. *maritima* L., and *P*. *media* L.) were examined with various assays that measure free radical scavenging ability: DPPH, hydroxyl radical, superoxide anion, and nitric oxide scavenger capacity tests, reducing power (FRAP) assay, and Fe^2+^/ascorbate induced lipid peroxidation [17], It can be hypothesized that this marked difference is largely attributable to the different solvent used for the extraction (80% methanol), but also to techniques (i.e. DPPH and FRAP) which tend to overestimate the effective antioxidant capacity of the polyphenols compared to the electrochemical system [44].

The antiproliferative activity found with the aqueous and hydro-ethanolic extracts of *P*. *lagopus* is likely due to the presence of acteoside, luteolin which are absent in our *P*. *major* extracts [15, 68, 69]. As previously reported, these two bioactive compounds had antiproliferative activity on different tumors such as primary colon cancer disease, skin cancer, breast cancer and brain tumor [70–72]. It is noteworthy to report that salicylic acid (SA, 2-hydroxybenzoic acid) was identified in the roots of *P*. *lagopus* but not in those of *P*. *major*. This component is endowed, apart its tolerance to biotic and abiotic stress in plants, with anti-inflammatory, analgesic, antifungal and antiproliferative properties [73]. Indeed, a significant cytotoxic effect of salicylic acid-containing ionic liquids towards human CaCo-2 cell lines was demonstrated [74]. In our case, we suggest that salicylic acid could act synergistically with other phenolic compounds in root extracts, thereby conferring this anti-Caco-2 activity. Plantamajoside was detected in all extracts from *P*. *lagopus* leaves and roots. According to some studies, plantamajoside isolated from *P*. *asiatica* inhibited the proliferation of two acute myeloid leukemia cells, MOLM-13 and HL-60, by enhancing apoptosis and inducing cell arrest in the G0/G1 phase [75, 76].

The antibacterial results agree with those of Sharifa et al. that, by Soxhlet- extracting the whole dried *P*. *major* plant with water, ethanol, or methanol, reported that the water extract was ineffective on *S*. *aureus* and *E*. *coli* as were the ethanolic and methanolic ones for *B*. *subtilis*. On the other hand, methanolic and less ethanolic extract inhibited effectively *S*. *aureus* and *E*. *coli*. [77]. According to Pesantes-Sangray et al., ethanolic extracts of *P*. *major* have an antibacterial effect *in vitro* on *Porphyromonas gingivalis* with a sensitivity of 75% and 100% and a minimal inhibitory concentration (MIC) of 50%, which made possible to estimate the use of these extracts in coadjuvant therapy for periodontal treatment [26]. The present used extracts had negligible to no activity against *Candida albicans*, in agreement with the results reported by Hassawi and Kharma, who tested leaf ethanolic extracts (95% EtOH) of *P*. *lanceolata* and *P*. *major* [78]. Similar results were attained by Sharifa et al., where aqueous, ethanolic or methanolic extracts of *P*. *major* did not inhibit the growth of *C*. *albicans* and *C*. *tropicalis* [77]. Orhan et al. by studying antibacterial properties of 21 plants, reported the MIC values of *P*. *major* leaf and flower aqueous extracts attained by decoction for 30 min [79]. Decoction was used as the extraction protocol in accordance to folk medicine then, dry matter was attained following freeze-drying of the extract. The inhibition activity was performed by dissolving the dry residue in 30% DMSO and 70% water and MIC values for *K*. *pneumonia*, *P*. *aeruginosa*, *S*. *aureus*, and *C*. *albicans* were 128, 64, 32, and 16 μg/mL, respectively [79].

In another report, ethanol and acetone extracts were attained from *P*. *major* leaves and evaluated against a board range of Gram+ and Gram- bacteria [80], among them some tested in the present research. Concerning MIC values reported for *E*. *coli* they resulted significantly different compared to our results (42.500 mg/mL). While, acetone extracts resulted more active against *B*. *ceureus* and *E*. *coli* with a MIC = 3.562 and 14.250 mg/mL, respectively. The same MIC value of *E*. *coli* was reported for *K*. *pneumonia* and *S*. *aureus* while, for *B*. *subtilis* and *P*. *aeruginosa* the value was 28.500 mg/mL. According to Sharifa et al., the MIC of ethanolic leaf extracts for *S*. *aureus* was 200 mg/mL and for *E*. *coli* 150 mg/mL [77]. The potential of some species of the *Plantago* genus is very well known; holding the example of Ferrazzano et al., where they noted that extracts of *P*. *lanceolata* at concentrations ranging from 2 mg/mL to 250 mg/mL had antimicrobial activity affecting the viability of a broad range of tested Streptococcus strains (*S*. *mutans*, *S*. *bovis*, *S*. *mitis*, *S*. *sobrinus*, *S*. *parasanguinis* and *S*. *viridans*) [81]. Therefore, these extracts could present a natural anti-cariogenic agent via an antimicrobial effect and could be useful as an auxiliary measure to control the proliferation of the cariogenic flora. Hydro-ethanolic extracts of the two species are rich in polyphenols. The antibacterial activity of the hydro-ethanolic extracts of the two species can be attributed to the compounds they contain such as plantamajoside and acteoside. Some studies have shown that acteoside possesses antibacterial and antifungal activity; for example, plant extracts containing verbascoside have been shown to enhance the antibacterial effect of gentamicin against *S*. *aureus* and *E*. *coli* [82]. Plantamajoside, isolated from *P*. *major*, has also been shown to be effective against *E*. *coli* (30 mg/mL) and *S*. *aureus* (50 mg/mL) [83].

Hydro-ethanolic extracts of the leaves and roots of *P*. *major* and *P*. *lagopus* contain flavonoids, namely luteolin and quercetin derivatives. The latter have previously been shown to exhibit antibacterial activity, suggesting that these compounds could be responsible for this observed activity [84]. Indeed, Studies have shown that luteolin and quercetin can inhibit the growth of *E*. *coli* and *K*. *pneumoniae* [85, 86]. In particular, rutin, a flavonoid similar to quercetin, has been found to exhibit strong inhibitory effects against *K*. *pneumoniae* and *E*. *coli*, with a MIC of 1024 μg/mL against *K*. *pneumoniae* and *E*. *coli*, respectively [86]. These findings suggest that polyphenols from leaves and roots of two species have the potential to be used as natural agents for combating *E*. *coli* and *K*. *pneumoniae* infections.

Among the reported MIC values there are great differences, this due to various factors among which the extraction protocols adopted (solvents used, extraction duration, temperature, DM/solvent ratio etc.), geo-pedological differences of plant origin as well as date of harvesting, still the reported trends are similar indicating the need to adopt standard experimental plans that will allow to make MIC values comparable and shed light on the role of the extraction protocols.

## 5. Conclusion

The identification of phenolic compounds of aqueous and hydro-ethanolic extracts of *P*. *lagopus* and *P*. *major* leaves and roots showed that polyphenols in the aqueous extracts of *P*. *lagopus* leaves and roots, are essentially flavonoids and phenolic acids. Electrochemical and analytical tests disclosed that the hydro-ethanolic fractions of the leaves of *P*. *lagopus* L. had an important antioxidant capacity, this was also noticed for the hot aqueous extracts of the roots of *P*. *major*. In addition, and for the first time aqueous and hydro-ethanolic extracts of *P*. *lagopus* leaves and roots showed significant antiproliferative activity mainly against the colorectal cancer line CaCo-2 after 12 h. This effect is likely related to the richness of *P*. *lagopus* extracts in phenolic compounds. Furthermore, the hydro-ethanolic extracts of *P*. *lagopus* and *P*. *major* have antibacterial effect against Gram- bacteria *E*. *coli* and *K*. *pneumonia* at a low concentration. The two species studied have significant antioxidant, antiproliferative and bacteriostatic potential and could be exploited as a source of natural antioxidant, anticancer and antibacterial agents to treat oxidative stress related pathologies. Future prospects involve identifying the non determined (ND) compound(s) the ones responsible for these activities; however, the mode of action of these compounds remains to be determined. Additionally, *in vivo* studies will need to be undertaken.

## Supporting information

S1 File Contains all the supporting tables and figures. (DOCX)
