# Supplementary figures and images for: Phytochemical evaluation and exploration of some biological activities of aqueous and ethanolic extracts of two species of the genus Plantago L
Source: PLoS One. 2024 Feb 29;19(2):e0298518. doi: 10.1371/journal.pone.0298518 (PMC10903836; doi:10.1371/journal.pone.0298518)

Supporting Information








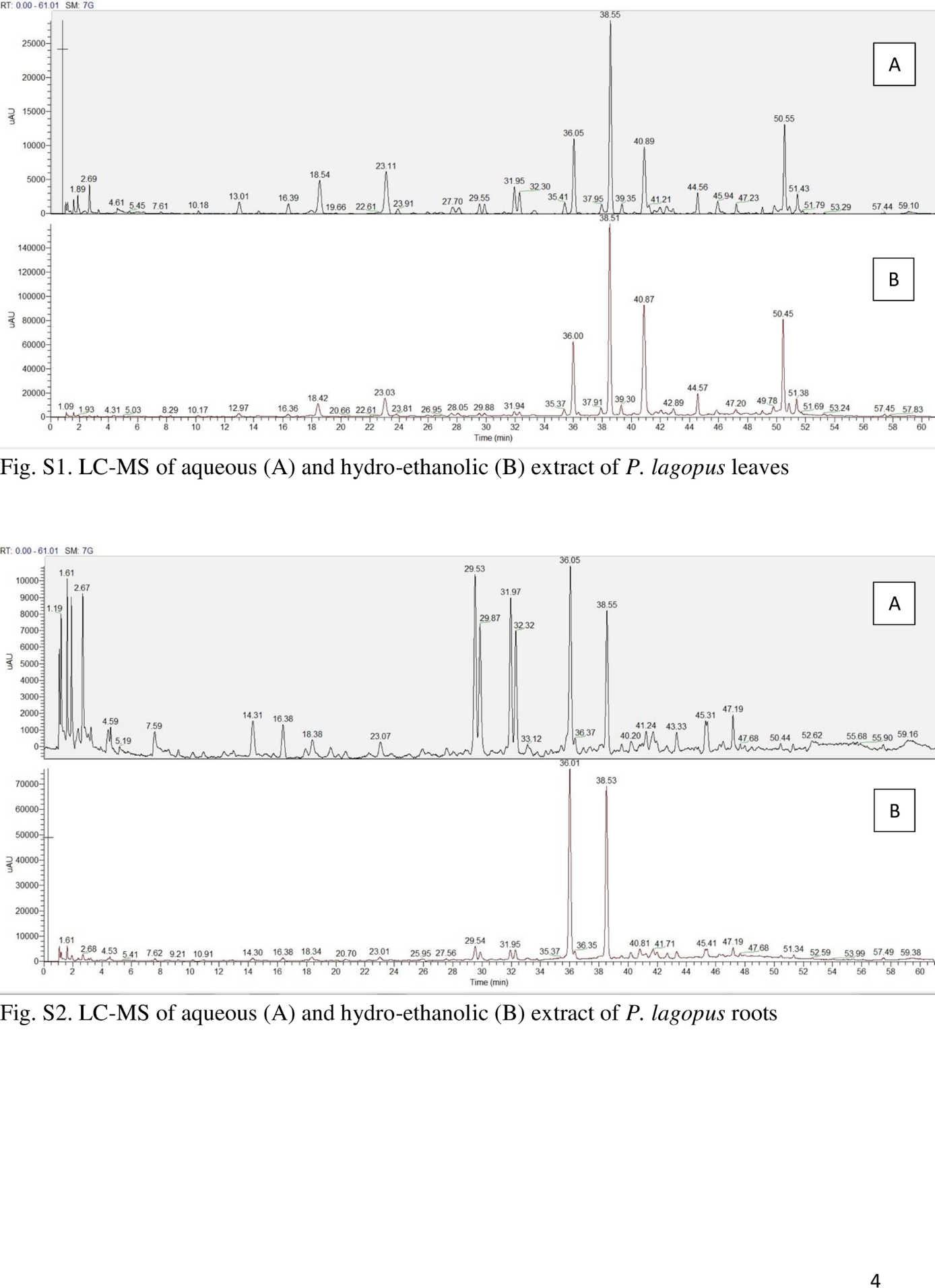

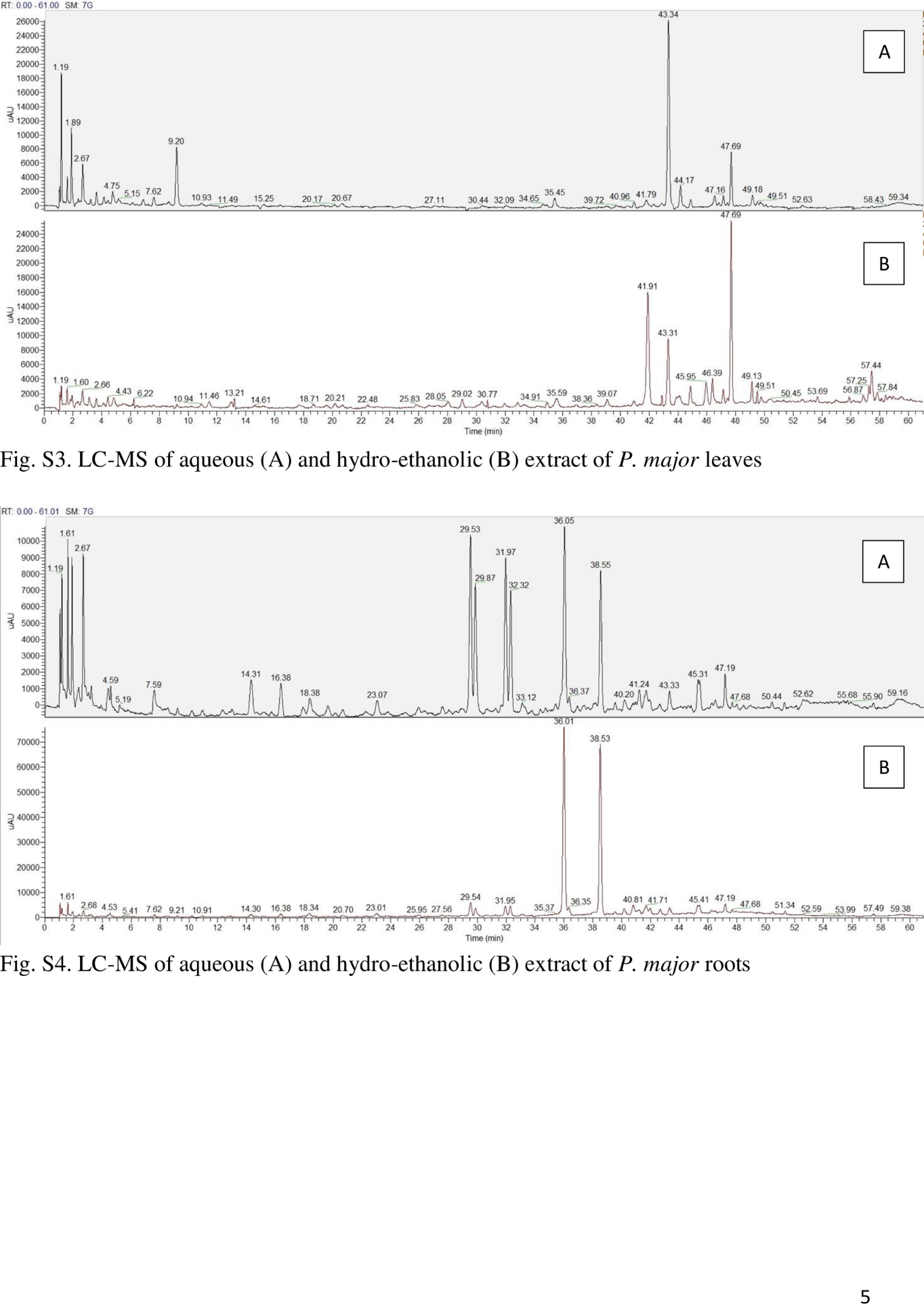

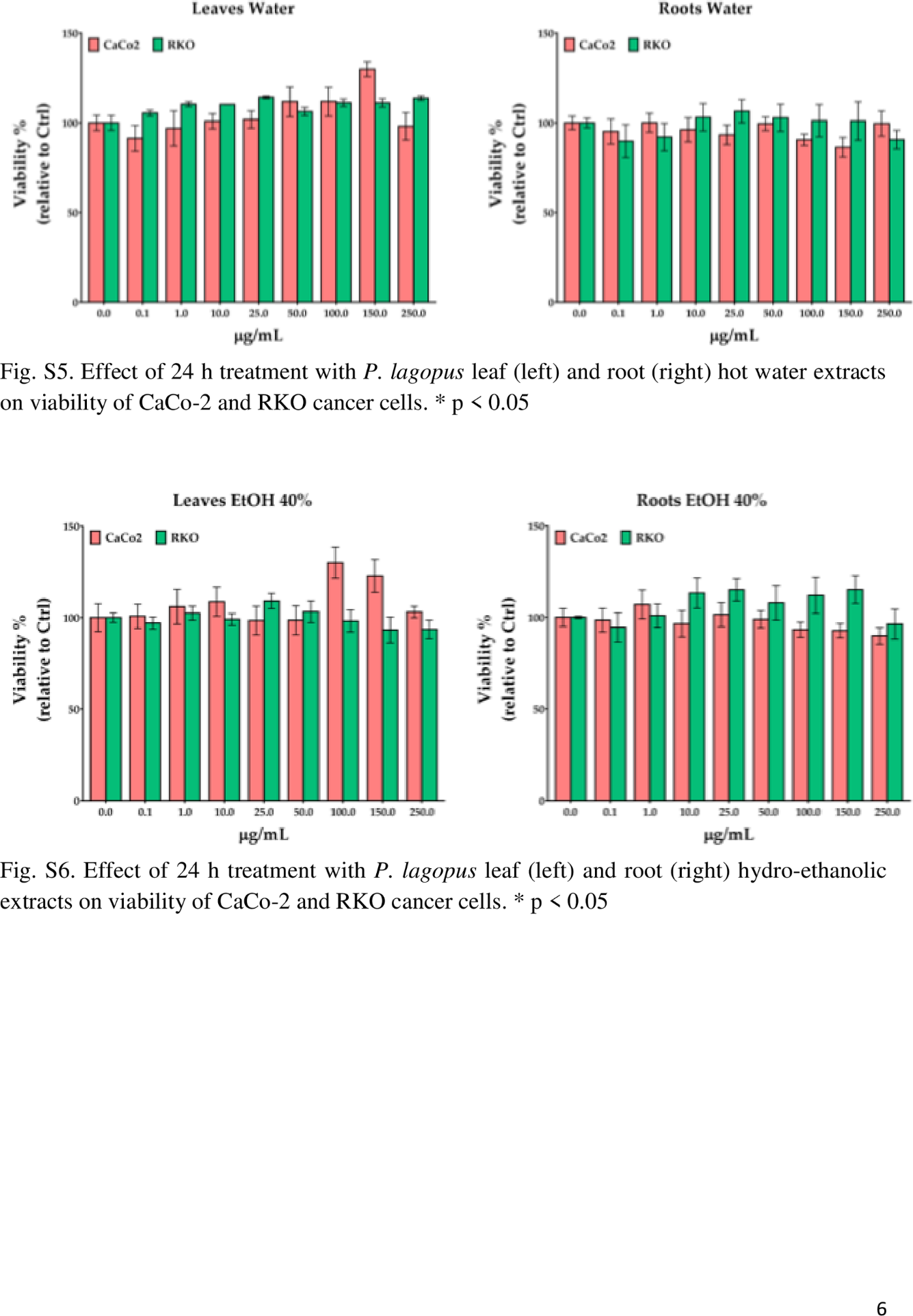

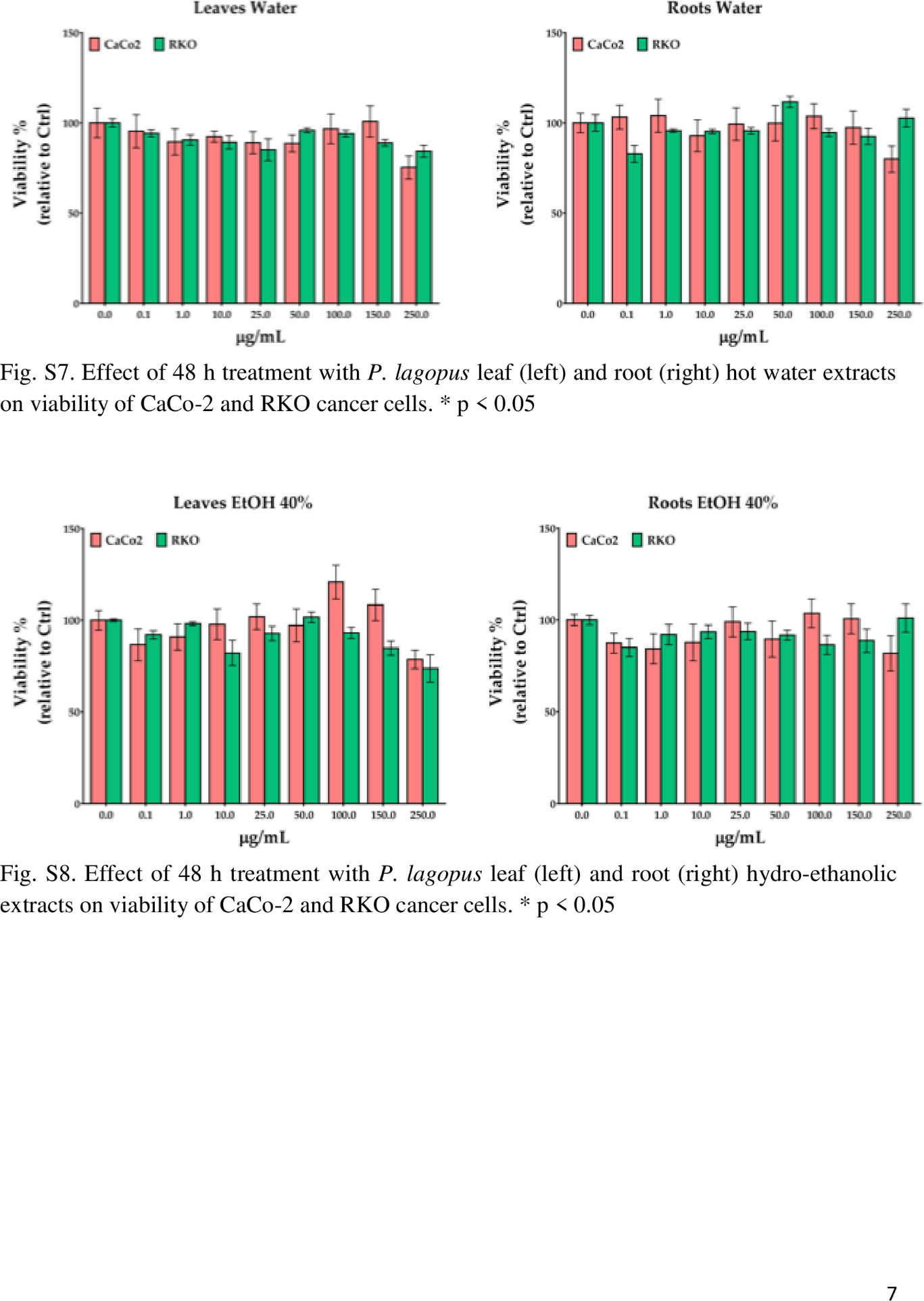

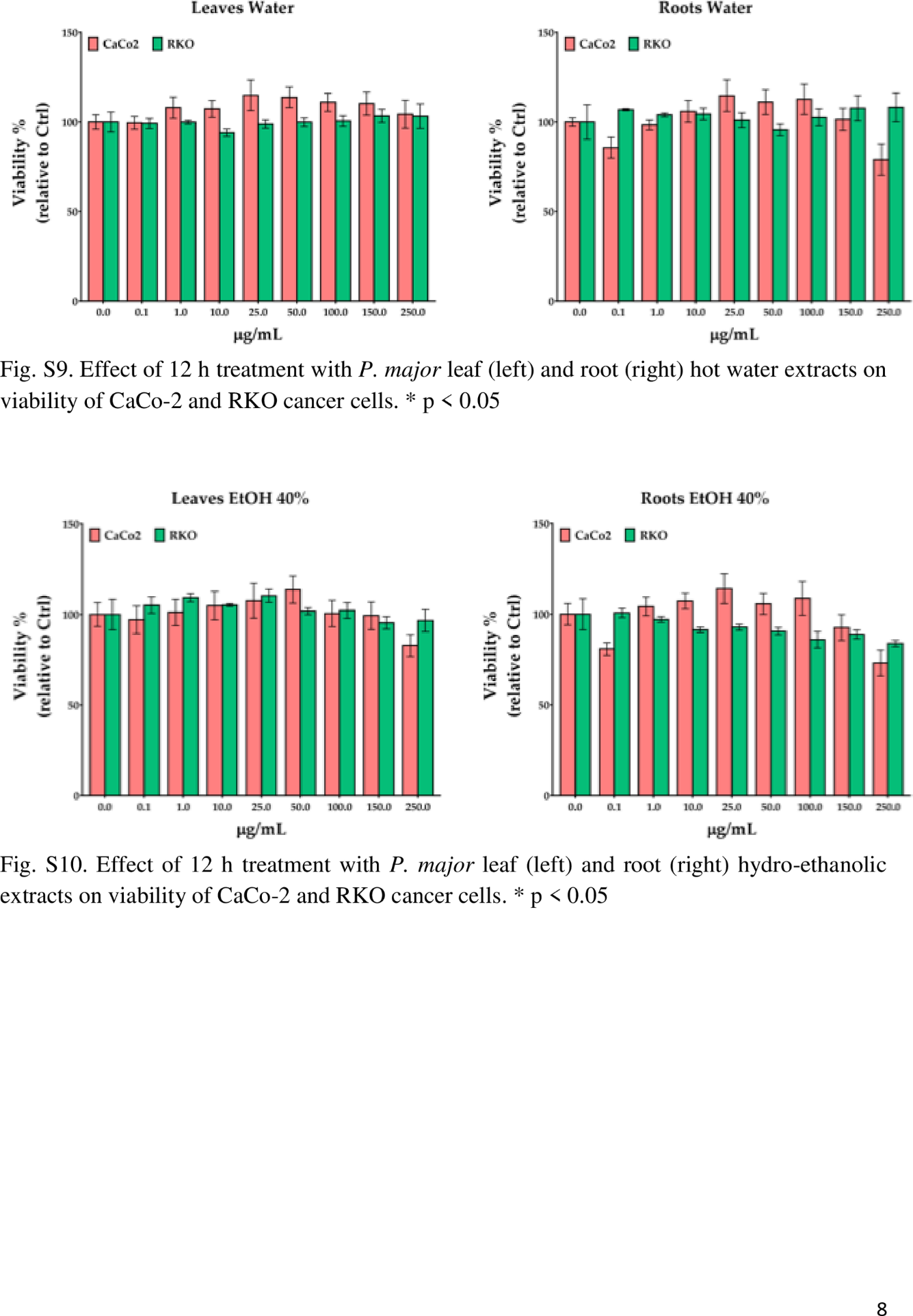

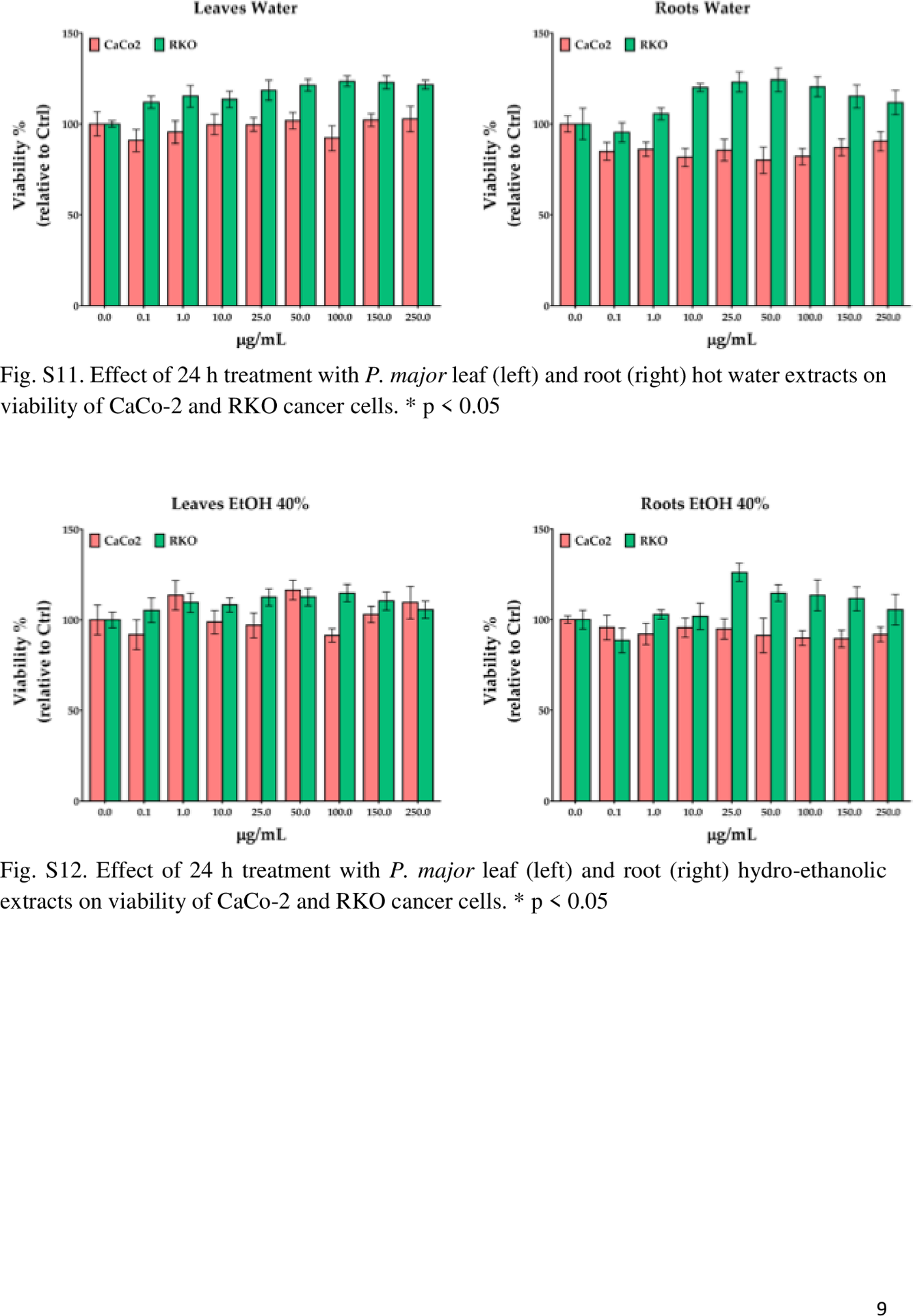

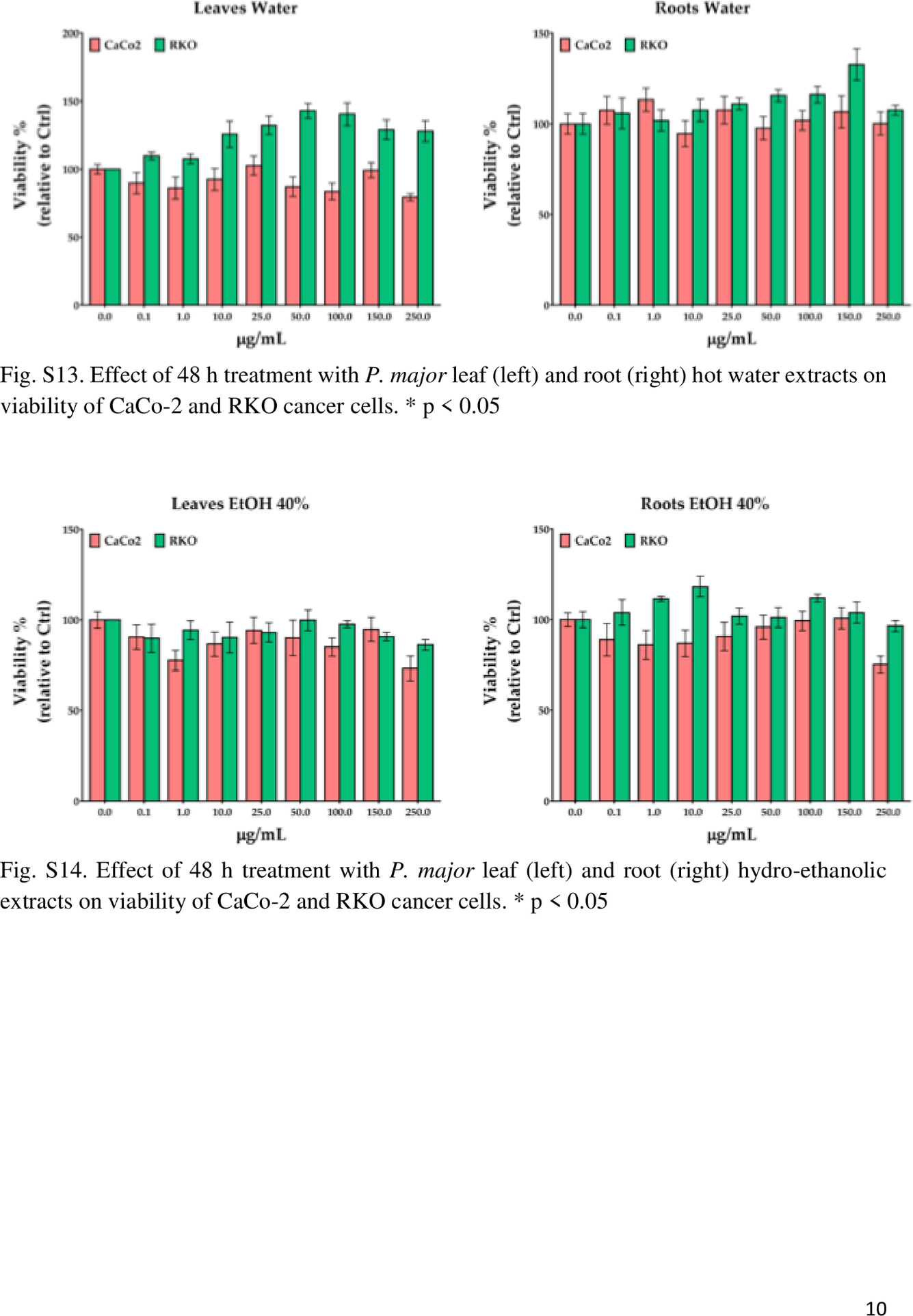

Supplement: S1 File — (DOCX) [file pone.0298518.s001.docx]
